# Antifungal Polymeric Materials and Nanocomposites

**DOI:** 10.3389/fbioe.2021.780328

**Published:** 2021-12-24

**Authors:** Winnie Ntow-Boahene, David Cook, Liam Good

**Affiliations:** ^1^ The Royal Veterinary College, Pathobiology and Population Sciences, London, England; ^2^ Blueberry Therapeutics Ltd., Macclesfield, England

**Keywords:** antimicrobial polymers, nanocomposites, antifungal, fungal infections, antifungal polymers

## Abstract

Rising global populations due to medicinal advancements increases the patient population susceptible to superficial and severe fungal infections. Fungi often implicated in these diseases includes the dermatophytes (*Microsporum spp*., *Epidermophtyon spp*., *Trichophyton spp*.) as well as species of the *Candida spp.*, *Aspergillosis spp*. and *Cryptococcus spp.* genera*.* In addition, increasing global populations leads to increasing agricultural demands. Thus, fungal infections of preharvested crops and stored food by plant pathogens such as *Magnaporthe oryzae* and *Fusarium oxysporum* can have detrimental socioeconomic effects due to food insecurity. Current antifungal strategies are based mainly on small molecule antifungal drugs. However, these drugs are limited by poor solubility and bioavailability. Furthermore, antifungal resistance against these drugs are on the rise. Thus, antimicrobial polymers offer an alternative antifungal strategy. Antifungal polymers are characterised by cationic and hydrophobic regions where the cationic regions have been shown to interact with microbial phospholipids and membranes. These polymers can be synthetic or natural and demonstrate distinct antifungal mechanisms ranging from fungal cell membrane permeabilisation, cell membrane depolarisation or cell entry. Although the relative importance of such mechanisms is difficult to decipher. Due to the chemical properties of these polymers, they can be combined with other antimicrobial compounds including existing antifungal drugs, charcoals, lipids and metal ions to elicit synergistic effects. In some cases, antifungal polymers and nanocomposites show better antifungal effects or reduced toxicity compared to the widely used small molecule antifungal drugs. This review provides an overview of antimicrobial polymers and nanocomposites with antifungal activity and the current understanding of their antifungal mechanisms.

## Introduction

Globally, fungal diseases significantly contribute to the increasing morbidity and mortality rates caused by infectious pathogens. Although fungal infections are usually superficial in healthy individuals, superficial infection often leads to more severe invasive infections in immunosuppressed patients (Kaushik, Pujalte and Reese, 2015). Superficial infections are commonly caused by fungal pathogens of the dermatophyte group (*Trichophyton spp*., *Epidermophyton spp*., *Microsporum spp.*) and includes onychomycosis (fungal nail), tinea corporis (fungal skin) and tinea pedis (athlete’s foot) (Kaushik, Pujalte and Reese, 2015).

In contrast, invasive fungal infections in immunosuppressed patients results in more severe consequences and can lead to tissue damage, organ failure and death (von Lilienfeld-Toal et al., 2019). Fungal pathogens commonly associated with invasive fungal infections include *Aspergillus spp.*, *Candida spp.*, *Cryptococcus neoformans* and *Fusarium solani* (Enoch et al., 2016). Currently, the rates of invasive infections are increasing, in part due to the increased lifespan of immunosuppressed patients and the use of immunosuppressive treatments (von Lilienfeld-Toal et al., 2019; Suleyman and Alangaden., 2016). It is estimated that the incidence of invasive fungal infection is ∼ 6 per 100,000 people (von Lilienfeld-Toal et al., 2019). However, this estimate is thought to be lower than the true rate due to poor diagnoses of invasive fungal infections (Denning et al., 2017). Indeed, the severity of invasive infections is directly dependent on the severity of immunosuppression (von Lilienfeld-Toal et al., 2019). Furthermore, fungal infections of preharvested crops such as cereals, grains, fruits and vegetables as well as their storage is a potential threat to food security as the global population is expected to be 9.8 billion by 2050 (Fisher, Gow and Gurr, 2016; United Nations, 2017). Thus, there is increasing demand for the production of good quality agricultural crops in high yield. These agricultural crops are susceptible to infection by *Candida spp., Fusarium oxysporum* and *Magnaporthe oryzae*; so antifungals have long been adopted within agricultural settings to maintain crop yield (Fisher, Gow and Gurr, 2016; Brauer et al., 2019). The main class of antifungals used in large-scale production and crop storage is the azole class where triazoles are the most commonly used (Brauer et al., 2019). However, the use antifungals within agricultural settings has direct implications in human health and environmental homeostasis due to increased risk of antifungal resistance (Fisher, Gow and Gurr, 2016; Brauer et al., 2019).

Existing antifungal drugs for the treatment of human infections include a limited number of chemical classes (polyene, allylamine, azole and echinocandin). These mainly target ergosterol within the fungal cell membrane or the synthesis of 1,3-β-D-glucan within the fungal cell wall. The treatment of superficial infections are associated with relapses due to improper clearance of infection (Campoy and Adrio, 2017). Thus, superficial infections often progress into chronic infections (Gupta et al., 2018). In addition, clinical applications of these drugs for invasive infections are often limited by their associated toxicities and complex intravenous administrations (Campoy and Adrio, 2017).

The emergence of single and multi-drug resistant fungi further compounds this treatment problem. Azole resistance amongst *Candida spp*. and *Aspergillus spp.* is on the rise along with echinocandins *C. glabrata* and multidrug resistant (azole, polyene, and echinocandin) *C. auris* (Perlin et al., 2017).

The limited chemical space and targets sites inhibited by current antifungals is compounded by the rising resistance rates. Antifungal resistance mechanisms include the development of efflux pumps, reduced expression of drug targets or structural alterations of drug targets as well as biofilm development (Cowen et al., 2014; Perlin et al., 2017). These mechanisms are innate in fungal species with reduced susceptibility to antifungal drugs such as *C. auris* but can be acquired within susceptible species. Consequently, the use of antimicrobial polymers provides a potentially superior alternative to improve current antifungal strategies as summarised in Table 1.

**TABLE 1:** A generalised overview of the advantages and disadvantages of antifungal polymers in comparison to small molecule antifungals.

	Compound	Advantages	Disadvantages	Reference
Antifungal polymers	Chitosan	Sustained drug delivery	Expensive to produce	Anitha et al., 2014
		High mucoadhesion	Poor potencies	Fan et al., 2018
		Oral delivery		Jung et al., 2020
		Low toxicity		Kurniasih et al., 2018
		Biocompatible		Mi et al., 2018, Tabriz et al., 2019
		*In vivo* biodegradation		Wei et al., 2019, Zhang et al., 2019
		High aqueous solubility		
		Easily chemically modified for flexible applications		
		Broad spectrum activity		
	PHMB	Sustained drug delivery	Some absorption in kidneys and liver of mice following oral ingestion	Elsztein et al., 2011
		Low toxicity		Muller & Kramer., 2008, Worsley et al., 2019
		Biocompatible		
		Most orally ingested PHMB in mice is excreted via urine and faeces		
		High aqueous solubility		
		Flexible applications		
		Broad-spectrum activity		
		No observed acquired resistance		
		No signs of liver		
	poly-ε-lysine (ε-PL)	Low toxicity	Low production yield and high production cost	Zhu et al. (2016)
		Biocompatible		
		Biodegradable		
		High aqueous solubility		
		Flexible applications		
		Broad-spectrum activity		
Antifungal drugs	Azole	Some azoles penetrate into the CSF	Poor aqueous solubility	Campoy and Adrio, 2017, Chowdhary et al., 2018, Perlin et al., 2017
		Topical or systemic delivery	Varying levels of toxicity	
		Broad-spectrum activity	*C. krusei, C. glabrata and A. fumigatus* show inherent or acquired resistance	
	Polyenes	Broad-spectrum activity	Poor aqueous solubility	Campoy and Adrio, 2017, Robbins et al., 2016
		Low incidence of resistance	Kidney, liver and infusion related toxicity	
	Allylamines	Terbinafine has a high oral absorption and long half-life	Poor aqueous solubility	Campoy and Adrio, 2017
		Lipophilic	Associated with recurring infections	
	Echinocandins	Low toxicity	Poor aqueous solubility	Campoy and Adrio, 2017, Perlin et al., 2017
		Better patient outcomes compared to azoles	Can only be given intravenously	
		Broad-spectrum activity	*C. glabrata* shows acquired resistance	
	Nucleoside analogs (5-Flucytosine)	Synergistic in combination with ketoconazole and amphotericin	Poor aqueous solubility	Campoy and Adrio, 2017
		Can penetrate the CNS, eye and urinary tract	Limited to yeasts	
			Some yeasts are resistant	
			Bone marrow and liver associated toxicity	
			Cannot be used alone	

Antimicrobial polymers are synthetic or natural organic compounds that exert a broad spectrum cidal effect or growth inhibition of microorganisms. These polymers have broad applications which include eye drops, wound irrigation solutions, wound dressings, cosmetics and water treatment. They also show excellent antibacterial and antifungal properties (Jain et al., 2014), but existing reviews are often focused on the antibacterial mechanisms of these polymers (Kamaruzzaman et al., 2019). In contrast, nanocomposites are hybrid materials and generally refer to a polymer matrix with the addition of nanoparticles. Nanocomposites can refer to colloids, gels, ceramic and metal matrices. The antimicrobial effects of nanocomposites are usually provided via the addition of antimicrobial nanoparticles or metal ions. The addition of these can sometimes improve the electrical and mechanical properties of these materials to widen their potential applications.

Given the burden of fungal diseases and limited treatment options; it is important to highlight the distinct antifungal mechanisms of antimicrobial polymers and nanocomposites to conventional antifungals. Thus, they may help to overcome the challenges of emerging antifungal resistance and to overcome or avoid the challenges of emerging antifungal resistance and biofilm development (Nobile and Johnson, 2015).

In some cases, antifungal polymers and nanocomposites display more potent antifungal effects relative to conventional antifungal drugs. Which begs the question, “Do antifungal polymers and nanocomposites have the potential to replace antifungal drugs?”. This review provides an overview of cationic antimicrobial polymers and nanocomposites with antifungal activity and the current understanding of the antifungal mechanism of action.

## Antimicrobial Peptides With Antifungal Activity

Antimicrobial peptides (AMPs) also known as host defence peptides are part of the innate immune response. They are produced by plants, animals and microorganisms and protect the host from invading pathogens (Fernández de Ullivarri et al., 2020). These peptides are amphiphilic with short sequences that are typically less than 100 amino acids (Fernández de Ullivarri et al., 2020). Most AMPs bear a cationic charge where histidine rich AMPs show strong antifungal activity (Kumar, Kizhakkedathu and Straus, 2018). An example of this are Cathelicidins. This class of antifungal AMPs are part of the human innate immune system and are primarily stored in lysosomes of macrophages (Scheenstra et al., 2020). However, there are some AMPs with anionic charges which require metal ions for biological activation (Harris et al., 2009; Phoenix et al., 2013). Anionic AMPs bind metal ions to form cationic salt bridges with anionic microbial membranes for membrane permeabilisation. Although this mechanism is attributed to some anionic AMPs, knowledge of their antimicrobial activity is still limited in comparison to cationic AMPs (Harris et al., 2009).

The main antimicrobial mechanism attributed to cationic AMPs is cell membrane disruption following electrostatic attraction interaction with anionic membranes. In addition, some AMPs can also act translocate across the membrane to act on intracellular targets for DNA and protein synthesis inhibition (Kumar, Kizhakkedathu and Straus, 2018). Despite the antimicrobial effects of AMPs, they are unstable with a short half-life (Kumar, Kizhakkedathu and Straus, 2018). Currently, there are 1,211 peptides of natural, semi synthetic or synthetic origin with antifungal properties in the antimicrobial peptide database (Wang et al., 2016).

### Synthetic Antimicrobial Peptides With Antifungal Activity

Synthetic peptide-like polymers are synthesised based on structural moieties of AMPs and therefore usually exert killing or growth inhibition of microbes via their cationic surface charge (Muñoz-Bonilla and Fernández-García, 2012). This class includes polymers with inherent antimicrobial activity such as polymers with quaternary nitrogen groups, halamines or poly-ε-lysine (ε-PL). Despite their excellent antifungal efficacy, many synthetic AMPs are not used clinically due to their potential toxicities in humans. Consequently, there is ongoing research for the design and optimisation of new synthetic AMPs with maintained significant antimicrobial efficacy but reduced toxicity.

Ramamourthy et al. (2020) synthesised peptides with varying number of lysine and tryptophan repeats (KW_n_-NH_2_) and their antifungal activity against *C. albicans* was investigated. The antifungal and biofilm eradication activities of these peptides increases with peptide length; where the shortest peptide KW_2_ displayed no antifungal activity and the longest peptide KW_5,_ displayed toxicity in a human keratinocyte cell line (Ramamourthy et al., 2020). The KW_4_ peptide did not disrupt fungal cell membranes. However, laser-scanning confocal microscopy showed KW_4_ localisation within the cytosol of *C. albicans* where it was bound to fungal RNA. This suggests the antifungal mechanism of these peptides is not entirely membrane permeabilisation. Instead, these synthetic AMPs enter fungal cells and localises within the cell where they inhibit cellular functions by binding to RNA and DNA resulting in fungal cell death (Ramamourthy et al., 2020).

Dodou Lima et al. (2020) synthesised novel synthetic AMPs with antifungal activity based on the structural determinants of antimicrobial peptides (pilosilin and ponericin) acquired from Dinoponera quadriceps (giant ant) venom. Pilosulin-like (Dq-2562 and Dq-1503) and ponericin-like (Dq-3162) peptides were the most potent antifungal peptides against *C. albicans*, *C. tropicalis*, *C. parapsilosis* and *C. krusei*. These synthetic peptides in combination with antifungal drugs (amphotericin B, fluconazole, miconazole, cyclopyrox, and nystatin) also showed synergistic effects and with low haemolytic activity (Dodou Lima et al., 2020).

Similarly, (Kodedová et al., 2019), developed synthetic antimicrobial peptides (lasioglossins, halictines and hylanines) based on the antimicrobial peptides of bee venom. These peptides were found to rapidly permeabilise the membranes of *Candida* species (C*. albicans, C. glabrata, C. parapsilosis, C. tropicalis, C. krusei* and *C. dubliniensis*) and *Saccharomyces cerevisiae*. However, species susceptibility to permeabilisation was dependent on the fungal membrane lipid composition. *C. glabrata* was observed to have an increased resistance to these synthetic peptides following pre-treatment with terbinafine to reduce fungal membrane ergosterol (Kodedová et al., 2019). The cationic peptides showed strong electrostatic interaction with the anionic membrane lipids (phosphatidylglycerol, phosphatidic acid and cardiolipin) and partly with phosphatidylinositol and the neutral phosphatidylethanolamine (Kodedová et al., 2019) which allows for better fungal selectivity. Thus, synthetic AMPs and peptide-like polymers based on the structural and functional properties of AMPs can overcome the limitations of natural AMPs whilst maintaining or improving their antifungal mechanisms.

Despite synthetic AMPs showing broad-spectrum antifungal activity and low toxicity, research into antimicrobial polymers is often focused on synthetic polymers as they are considerably cheaper to produce in comparison and share functional cationic similarities with AMPs.

### Synthetic Antimicrobial Peptide-Like Polymers With Antifungal Activity

Nylon-3 copolymers or poly-β-peptides are a family of synthetic peptide-like polymers based on the structural moiety of AMPs and are made via anionic ring-opening polymerisation of β-lactams (Yang et al., 2011). The resulting polymers (MM-TM, DM-TM, and NM) display improved potency against a diverse range of fungal species including *C. albicans, C. krusei, C. auris, C. neoformans,* C. amylolentus and *A. fumigatus* with improved fungal selectivity over mammalian cells (Liu et al., 2013; Liu et al., 2014; Rank et al., 2018). The most effective nylon-3 polymer was found to be poly-βNM, composed of the cationic subunit βNM and the hydrophobic subunit CH in differing proportions. It shows potency against both planktonic, biofilm and mature biofilm *C. albicans K1* with an MIC of 3 μg/m (Liu et al., 2015). Polymers with a higher βNM content/cationic charge density are more effective at biofilm inhibition and display more potent fungicidal effect relative to amphotericin B (AmpB). Interestingly, these polymers with increased cationic charge density displayed reduced activity against 48-h biofilm (Liu et al., 2015). This reduction may be due to anionic biofilm components such as DNA, RNA and proteins which may bind to the polymer to hinder penetration to intracellular antifungal sites. Fortunately, certain synthetic peptide-like polymers are unlikely to be toxic and show great safety profiles. For example, the polyamide backbone of the nylon-3 polymers is thought to improve biocompatibility as it is protein-like (Frackenpohl, Arvidsson, Schreiber and Seebach, 2001).A more hydrophobic version of this polymer (70:30, βNM:CH) shows significant haemolysis at 2 mg/ml (Liu et al., 2015). However, this is in keeping with observed toxicities of hydrophobic regions of polymers (Cuervo-Rodríguez, Muñoz-Bonilla, López-Fabal and Fernández-García, 2020).

## Synthetic Antimicrobial Polymers With Antifungal Activity

Antimicrobial polymers show cidal effects in both yeasts and filamentous fungi as their cationic charge lends a high binding affinity to the negatively charged microbial membrane surface as summarised in Table 2. These polymers have gained an increase attention in antifungal research as it minimises the challenge of discovering novel antifungal targets and expands the potential applications of new and existing antifungal agents. They are neither costly or labour intensive to manufacture, can easily be chemically modified for broader applications and integrated into nanocomposites for controlled release (Gibney et al., 2012). Subsequently, it has led to interest in developing novel antimicrobial peptides with improved physicochemical properties.

**TABLE 2:** Summary table of synthetic antifungal polymers and their reported targets.

Antifungal polymer	Membrane	Cell wall	Intracellular targets	Toxicity	Reference
Synthetic antimicrobial peptides	Does not appear to permeabilise fungal cell membranes	—	Binds to nucleic acids	Dependent on hydrophobic region length	Ramamourthy et al. (2020)
Synthetic antimicrobial peptides	Membrane permeabilisation	—	Likely	Low haemolytic activity	Dodou Lima et al. (2020)
(Nylon-3 copolymers)	Membrane permeabilisation	—	—	Low toxicity, dependent on hydrophobic region length	Liu et al., 2015
					Rank et al. (2018)
PHMB	Membrane permeabilisation	Cell wall target	Nucleus, binds to DNA/RNA	Low	Elsztein et al., 2011
POGH	Membrane permeabilisation likely	—	Likely	Slightly	Luo et al. (2017)
PQ-1	Membrane permeabilisation	Prevents conidia germination	Likely	—	Codling., 2003
					Kilvington et al. (2013)
PEI	Membrane permeabilisation	—	Likely	Slightly, dependent on hydrophobic region length	Azevedo et al. (2014)
			Cell membrane depolarisation, binds to nucleic acids		
Chitosan	Membrane permeabilisation	—	Nucleus, binds to DNA/RNA	Low toxicity	Palma-Guerrero et al., 2008
PHMB derivatives (PHMG-P PHMGH)	Membrane permeabilisation	Cell wall target	Likely	Low toxicity but severe toxicity when inhaled	Choi, Kim and Lee, (2017)
					Olmedo et al. (2018)
(N-(2-hydroxypropyl)-3-trimethylammonium chitosan chlorides) HTCC	Membrane permeabilisation	—	Likely	Low toxicity	Hoque et al. (2016)
Quaternary ammonium chloride derivatives of chitosan	Membrane permeabilisation	—	Likely	Low toxicity	Jung et al. (2020)

### Polyhexamethylene Biguanide

Polyhexamethylene biguanide (PHMB) is a synthetic quarternary ammonium polymer which has been established to be an effective antimicrobial agent with added advantages of low toxicity (Behrens-Baumann et al., 2012; Walls et al., 2013; Sanada et al., 2014; Worsley et al., 2019). It exhibits a high therapeutic index and broad-spectrum antifungal activity due to its biguanide groups and is commonly used as a preservative in cosmetics, water purifications systems and contact lense cleaning solutions. It is also used clinically for wound cleaning where it shows excellent biocompatibility (Gilbert et al., 2001; Muller & Kramer., 2008). Although PHMB shows membrane disruption abilities due to its phospholipid binding (Chindera et al., 2016), the exact antifungal mechanism of action remains unclear. The antifungal mechanism is thought to involve cell wall destabilisation and membrane permeabilisation. Gene expression studies in *S. cerevisiae* indicated an increase in the expression of cell wall integrity genes and protein kinase C which regulates cell maintenance. This suggests PHMB also damages the b-glucan structure of the *S. cerevisiae* cell wall (Elsztein et al., 2011).

### Polyoctamethylene Guanidine Hydrochloride

Similar to PHMB, Polyoctamethylene guanidine hydrochloride (POGH) is also used as a disinfectant. It has known antibacterial properties but knowledge of its effect on eukaryotic membranes and subsequently fungi is limited. It shows haemolytic activity with an LD50 range of 500–5,000 mg/kg which indicates slight toxicity. Increasing the hydrophobic alkyl carbon chain lengths of this polymer is accompanied by an increase in biocidal and haemolytic activity (Luo et al., 2017). Large unilamellar vesicles loaded with calcein dye showed an increase in polymer/Palmitoyl oleoyl phosphatidyl choline (POPC) membrane interaction and dye leakage rate. Like its analogues, POGH disrupts phospholipid membranes of the eukaryotic cell (Luo et al., 2017).

### Polyquartenium-1

Polyquartenium-1 (PQ-1) or polidronium chloride is another quartenary ammonium antimicrobial polymer used in eye contact lense cleaning solutions. Though PQ-1 is known to have predominantly antibacterial properties, it was also found to induce K+ leakage in *C. albicans* and *A. fumigatus*, which indicates membrane damage. Furthermore, it caused lysis of spheroplasts of *S. marcescens*, but not those of *C. albicans* (Codling, 2003) and prevents the germination of *F. solani* conidia by a 3 to 4 log_10_ fold reduction (Kilvington et al., 2013).

In contrast to PHMB and related polymers, PQ-1 is considered too large to enter mammalian cells and lacks hydrophobic regions (Ammar, Noecker and Kahook, 2010). This is made evident by PQ-1’s observed ability to intercalate within the biomembrane structure of small unilamellar vesicles whereas PHMB adsorbs unto the biomembrane surface (Horner et al., 2015).

### Polyethylenimines

Polyethylenimines (PEI) are polymers with an amine group and a two carbon (CH_2_CH_2_) spacer. They exist in linear and branched polymeric forms with different states at room temperature (Yemuland and Imae, 2008). Linear PEI with secondary amine groups are solid at room temperature whereas branched versions of this polymer with primary, secondary and tertiary amine groups are liquid at room temperature. PEIs are also membrane permeabilisation agents of bacteria and can also enter cells (Yuan and Li, 2017). Thus, they have been used extensively in drug delivery and *in vitro* transfections. The exact antifungal mechanism is unclear, but PEIs have been shown to depolarise *C. albicans* membranes (Azevedo et al., 2014). PEI demonstrates antifungal activity for *C. albicans* with an MIC of 4.83 mg/ml and antibiofilm activity at concentrations above and including the (MIC, 2XMIC, 4XMIC) (Azevedo et al., 2014). Mammalian cells appear tolerant to low molecular weight PEIs. In comparison, high molecular weight PEIs are potentially more toxic to mammalian cells due to their increased cell membrane disruption (Gibney et al., 2012).

### Chitosan

Chitosan is a linear β-1,4-polysaccharide of D-glucosamine and N-acetyl-D-glucosamine made from the deacetylation of chitin; a component of the fungal cell wall and exoskeleton of crustaceans (Muxika et al., 2017). It is used as an antimicrobial agent in wound dressings and improves drug delivery across epithelial cell membranes (Anitha et al., 2014). It has applications as a fining agent in winemaking and in agriculture where it is used as a biopesticide to prevent fungal infections of plants as it enhances plant defense systems whilst exerting an antimicrobial effect (Maluin et al., 2020). Chitosan increases the production of the phytoalexin, pisatin and other defence genes including β-glucanase and chitinase (Lopez-Moya et al., 2019). Short chain chitosan, with ≤7 sugar residues are water soluble whereas long polymers of chitosan are only water soluble at pH 7 and below. This has further implications as the short chain chitosan is more effective against planktonic fungi whereas long chain polymers of chitosan show greater effect against fungal biofilms (Jung et al., 2020). In addition, the degree of deacetylation and molecular weight influences the antimicrobial activity of this polymer (Lopez-Moya et al., 2019).

As an antimicrobial, this cationic polymer has a strong binding affinity for nucleic acids and shows membrane permeabilisation ability which results in leakage of cell contents (Young et al., 1982). Chitosan is also able to enter cells where it inhibits growth and RNA synthesis in *F. solani* (Palma-Guerrero et al., 2008). Acid cleaved chitosan with shorter chains show more potency and have a lower MIC than native chitosan (Kendra and Hadwiger, 1984). However, not all fungi are susceptible to chitosan’s effects.

Some fungi are inherently resistant to chitosan as chitosan makes up a significant proportion of their cell wall which increases their tolerance (Kendra and Hadwiger, 1984). The cell membranes of these fungi also contain saturated phospholipids which decreases membrane fluidity and subsequently reduces membrane permeabilisation of chitosan. An N. crassa mutant with a reduced proportion of unsaturated fatty acids and phospholipids, showed a decrease in chitosan antimicrobial activity compared to wild type N. crassa (Palma-Guerrero et al., 2010). This study also showed that chitosan-resistant and -sensitive fungi belong to different fungal families (Palma-Guerrero et al., 2010).

Chitosan can be modified at the amino group or alcohol groups for phosphorylation, N-modified chitosan, O-modified chitosan, N,O-modified chitosan derivatives, N-alkyl or N-benzyl chitosan. These modifications can improve the solubility, reduce toxicity and increase the antifungal effects of chitosan (Fan et al., 2018; Kurniasih et al., 2018; Mi et al., 2018; Wei et al., 2018; Tabriz et al., 2019; Wei et al., 2019; Zhang et al., 2019).

## Derivatives of Synthetic Antifungal Polymers

### Polyhexamethylene Guanine Phosphate and Polyhexamethylene Guanidine Hydrochloride

Polyhexamethylene guanine (PHMG) is a cationic polymer closely related to PHMB and is often used in its salt derivatives (polyhexamethylene guanine phosphate (PHMG-P) and polyhexamethylene guanidine hydrochloride (PHMGH)) as a disinfectant. Polyhexamethylene guanidine hydrochloride (PHMGH) can prevent the formation of *C. albicans* biofilms on solid surfaces (Garcia et al., 2020). It also demonstrates a higher antifungal potency than AmpB and a lower toxicity profile with no haemolytic activity or release of lactate dehydrogenase within the concentration range of 1.25–40.0 μg/ml (Choi, Kim and Lee, 2017). The antifungal mechanisms of PHMG appears to involve the formation of pores within the fungal cell membrane. A membrane study using 1,6-diphenyl-1,3,5-hexatriene labelling showed a significant loss of *C. albicans* membrane phospholipids and K+ leakage after PHMGH exposure. Dextran leakage from large unilamellar vesicles was also observed following PHMG treatment with pore sizes ranging between 2.3 and 3.3 nm. (Choi, Kim and Lee, 2017). The uses of this polymer also include agriculture, where PHMG inhibits conidia germination at 5 mg/ml and growth at 50 mg/ml of *P. digitatum,* an important pathogenic fungus of plants. Besides cell wall and membrane disruption, PHMG adsorbs unto the conidial surface, resulting in conidia deformation and collapse (Olmedo et al., 2018). Despite its antifungal advantages, when aerolised for agricultural use and inhaled, it causes lung damage resulting in fibrosis by destroying the membranes of alveolar epithelial cells inducing epithelial-mesenchymal transition (EMT) (Park et al., 2019).

### N-(2-Hydroxypropyl)-3-Trimethylammonium Chitosan Chlorides

The chitosan derivative, N-(2-hydroxypropyl)-3-trimethylammonium chitosan chlorides (HTCC) is an antimicrobial polymer used as a preservative within the cosmetics industry. Hoque et al., 2016 showed HTCC to have potent antifungal activity (MIC = 125–250 μg/ml) with killing observed within 2 h. Like chitosan, it also targets the fungal cell membrane to increase membrane permeability and shows very low toxicity (HC50 = >10000 μg/ml) within a mouse model.

### Quaternary Ammonium Chloride Derivatives of Chitosan

Amphiphilic quarternary ammonium chloride derivatives of chitosan of differing hydrophobic alkyl chain lengths (C_4_, C_8_, and C_12_) were designed by Jung et al. (2020) with the aim of binding to fungal biofilms. Shorter alkyl chain polymers (C_4_) showed the most potency against planktonic *C. albicans* (Jung et al., 2020). However, in the treatment of *C. albicans* biofilms, longer alkyl chains (C_8_ and C_12_) were more effective at eradication (Jung et al., 2020). All the chitosan derivates showed good biocompatibility and low toxicity with rat skin fibroblast cells (L929 cell line) when the MTT assay was performed (Jung et al., 2020).

## Functionalised Antifungal Polymers

**FIGURE 1:**
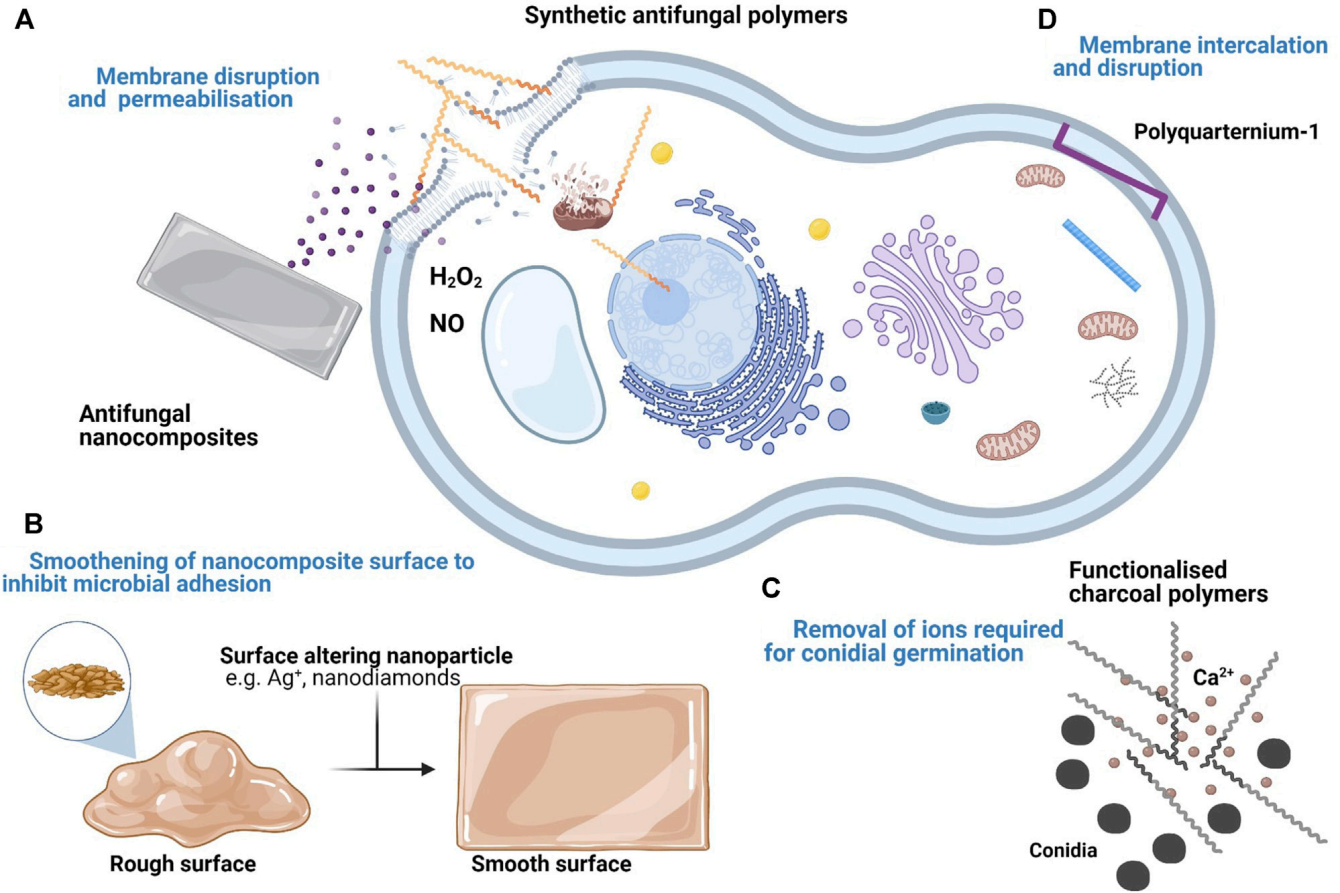
Summary of the proposed targets and mechanisms of antifungal polymers and nanocomposites. **(A)** Cationic polymers and their derivatives disrupt the fungal cell membrane. Upon entry into the fungal cell, they also disrupt organelle membranes and bind to DNA. Some antifungal nanocomposites with membrane disrupting compounds e.g. Chlorhexidine diacetate salt (CDA) also show a similar effect. **(B)** Some nanocomposites demonstrate an antifungal effect by forming deposits within grooves of the material surface to minimise fungal adhesion. **(C)** Inert polymers can be functionalised following the addition of antimicrobial compounds. Some functionalised polymers e.g. charcoal polymers inhibit fungal growth by removing essential ions (e.g. Ca2+) necessary for conidia germination. **(D)** Some antimicrobial polymers e.g. PQ-1 do not permeabilise the fungal cell membrane. Instead, they disrupt the membrane via intercalation.

Not all antimicrobial polymers have an inherent antimicrobial effect. Some inert biocompatible polymers are modified with functional groups which exert an antimicrobial effect. Figure 1 shows the varying antifungal mechanisms displayed by inherent and functionalised antifungal polymers. Ranging from membrane permeabilisation, membrane intercalation and ion sequestering. These functionalised polymers include polystyrenes, polypyridines and polymethylacrylates and do not usually show membrane permeabilisation. In other cases, polymers with inherent antimicrobial properties can also be potentiated through functionalisation.

### Functionalised Polymethacrylate Polymers

Polymethacrylates (PMMA) refers to any polymer comprised of a methacrylic acid ester with a wide use of applications. For example, it is used within the cosmetics industry as a wrinkle filler, in ophthalmology as intraocular lenses and in dentistry as dental material (Frazer et al., 2005; Fan et al., 2018). Therefore, it is highly compatible with human tissue and due to its medical applications in medical devices, it is at risk of microbial colonisation from pathogens such as *C. albicans*. Composites were made of PMMA with undecylenic acid (UA); a monounsaturated lipid with established antifungal activity at differing UA concentrations (3–12%) (Petrović et al., 2018). The addition of UA to PMMA altered the polymer’s surface to increase hydrophilicity. *C.albicans* exposed to the surface of these PMMA-UA composites showed reduced attachment, growth and increased death of these fungal cells at UA concentrations 6%. Although UA concentrations 9% showed 95% eradication of *C. albicans*, these composites were highly toxic to human cells with a 50% reduction in viability (Petrović et al., 2018). A balance must therefore be achieved between the ratio of inert polymer to active biocide to produce an effective composite with antifungal activity and lower toxicity.

Composites of PMMA and chlorhexidine diacetate salt (CDA), a cationic disinfectant used to clean the skin prior to surgery showed growth and biofilm inhibition of *C. albicans* and drug release over time (Maluf et al., 2020). This was observed at different CDA concentrations of 2, 1, and 0.5% with no significant differences in antifungal activity. Chlorhexidine creates pores in the polymer matrix as it diffuses out. This further facilitates diffusion from new to interconnected pores and allows maintained drug release levels from the polymer matrix (Maluf et al., 2020).

### Charcoal Polymers

An antifungal food packaging material made from charcoal polymers were developed by Yang et al. (2016). The charcoal polymers were developed by combining charcoal powders with plastic resin under a vacuum (Yang, Cha, Kim and Choi, 2016). These polymers were tested against filamentous fungi (*P. variotii*, C. globosum and *T. virens*). *P. variotii* and *T. virens* showed growth inhibition of 10 and 30% respectively following exposure. *C. globosum* conidia, pretreated with these polymers also showed no germination during 5 days of culture following exposure (Yang, Cha, Kim and Choi, 2016). The antifungal mechanism of these polymers involves the adsorption of Ca^2+^ into the nanosized pores of the charcoal. An estimated 15 mM of Ca^2+^ were removed from CaCl_2_ solution with 0.2 g/ml of polymers within 5 h (Yang, Cha, Kim and Choi, 2016). These polymers reduce the amount of readily available Ca^2+^ required for conidia germination and fungal hyphae growth.

### Metal Ion Functionalised Polymers

Metal ions, usually from the d-block of transition metal elements (V, Ti, Cr, Co, Ni, Cu, Zn, Tb, W, Ag, Cd, Au, Hg) are often used as antimicrobial agents as they are toxic to both microorganisms and their biofilms (Turner, 2017). Although studies into the efficacy of these ions are usually focused on bacteria, they also show antifungal effects. Therefore, functional antifungal polymers can be created from the incorporation of these ions into inert polymers. Three new Co^II^-coordination polymers (Co-CPs), also containing glutarates and bipyridyl ligands, were formulated [Co_2_(Glu)_2_(µ-bpa)_2_]·(H_2_O)_4_ (1), [Co_4_(Glu)_4_(µ-bpp)_2_] (2), and [Co_2_(Glu)_2_(µ-bpe)_2_]·(H_2_O)_0.5_ (3) (Kim et al., 2019). All three polymers showed more potency against the yeast *C. albicans* than the filamentous fungi *A. niger* conidia within the same treatment time with minimal leaching of Co^II^ ions (Kim et al., 2019). *C. albicans* cells appeared crushed and *A. niger* conidia appeared wrinkled with peeling outer layers. The antifungal mechanism of these ions includes the generation of reactive species NO and H_2_O_2_ which may play a role in fungal killing. For *A. niger*, it also seems to involve cell wall damage which was not observed in *C. albicans* and is likely due to difference in cell wall structure of the two fungal species (Kim et al., 2019).

## Antifungal Nanocomposites

Antimicrobial nanocomposites are materials that comprise nanosized antimicrobials or biocides to improve the antimicrobial effect. Nanocomposites show a wide range of applications due to a variety of materials and biocides that can be used in combination as shown in Table 3. In some cases, the addition of these nanocompounds improves the structural stability and even thermal stability of the nanocomposite (Norouzi, Zare and Kiany, 2015; Hossain et al., 2019).

**TABLE 3:** Summary table of functionalised antifungal polymers and their reported targets.

Antifungal polymer	Membrane	Cell wall	Other targets	Toxicity	Reference
PMMA-UA	Inhibits lipid biosynthesis	—	—	Toxic	Petrović et al. (2018)
PMMA- chlorhexidine diacetate salt (CDA)	Membrane permeabilisation	—	—	Low toxicity	Maluf et al. (2020)
Polymers functionalised with metal ions	—	Cell wall target	Low level Cytoplasmic generation of H_2_O_2_, NO	—	Kim et al. (2019)
Charcoal polymers	—	—	Binds to Ca^2+^ prevent fungal growth	—	Yang, Cha, Kim and Choi, (2016)

### Ag^+^ Based Antifungal Nanocomposites

Antimicrobial metal nanoparticles (NPs) are increasingly used as an alternative to antimicrobial drugs as it minimises the risk of developing antimicrobial resistance (Zare and Shabani., 2016). However, they are associated with toxicity (Grunlan, Choi and Lin, 2005). One approach to improve biocompatibility is to incorporate them into biomaterials such as proteins, peptides and sugars. A green synthesis approach by Jia et al., 2020 was used to generate Ag-Au alloy nanoparticles (Ag-AuNPs) with potential broadspectrum uses which includes as a coating material for medical devices or for drug delivery. The size of these NPs was dependant on Ag content; where increasing Ag, increased NP size. These nanocomposites showed good antifungal activity against *C. albicans* due to the silver ions. However, the Ag-Au alloy nanoparticles showed improved antifungal activity compared to the silver nanoparticles with MICs as low as 8 mg/ml (Jia et al., 2020). The Ag-Au alloy nanoparticles inhibited growth and biofilm development of fungi in a dose dependent manner with complete suppression and clearance at >16 mg/ml. MTT assays of RAW264.7, Hela and LO_2_ cells exposed to these nanocomposites showed no significant toxicity at concentrations as high as 128 mg/ml (Jia et al., 2020).

Although the antifungal mechanism(s) associated with AgNPs are unclear, Alghuthaymi et al. (2020) provides some understanding. Chitosan-silver nanocomposites (Ag-Chit-NCs) were developed against *P. expansum;* a fungal pathogen often associated with animal infections such as dairy cattle and the spoilage of agricultural crops and their storage. The nanocomposite was developed with silver content of 5.9 w/w%. Transmission electron microscopy (TEM) analysis showed 72% of the nanoparticles incorporated into the chitosan matrix were.

∼ 4 nm. Ag-Chit-NCs <10 nm at 0.3 mg/ml showed significant antifungal activity. These particles were observed to internalise within fungi to form cytoplasmic agglomerates (Alghuthaymi et al., 2020). The antifungal mechanism also appears to involve cell organelle and protein binding along with protein and DNA damage (Alghuthaymi et al., 2020). This is in keeping with work by Xia et al., 2016 which shows Ag^+^ nanoparticles damaged the fungal cell wall, cell membrane, mitochondria, chromatin, and ribosome.

As mentioned earlier under section 5.1, PMMA is a polymeric material frequently used in dental medicine and is therefore at risk of colonisation. Thus, the incorporation of AgNPs within prosthetic matrices could be beneficial to prevent their oral colonisation by microorganisms such as *C. albicans*. Nanocomposites were made of PMMA and citrate-capping AgNPs with a size of 20 nm and tested for *C. albicans* colonisation (De Matteis et al., 2019). *C. albicans* colonisation was analysed by the Miles and Misra technique and scanning electron microscopy (SEM) at 24 and 48 h. The nanocomposites showed a significant reduction in colonisation after 48 h of incubation. At 3.5 wt% AgNPs nanocomposite, the observed colony count was 0.41 × 10^6^ ± 0.43 cfu/ml whereas the control PMMA showed 7 × 10^6^ ± 1.03 cfu/ml (De Matteis et al., 2019). The addition of the AgNPs to PMMA filled the pores of the matrix causing a reduction in surface roughness of PMMA and introduced a negative charge to the polymer surface due to the citrate capping (De Matteis et al., 2019). The alteration of polymer surface topology and introduction of a negative charge reduced the adhesion of *C. albicans* to the polymer surface whilst the addition of Ag provided an antifungal effect.

Zhang et al. (2017) also tested the antifungal and cytotoxic effects of a developed silver bromide/cationic polymer (poly (4-vinylpyridinium) nano-composite (AgBr/NPVP)-modified PMMA-based dental resin against oral colonisation by *C. albicans* (Zhang et al., 2017). AgBr/NPVP was added to the PMMA resin at 0.1, 0.2, and 0.3 wt% with PMMA resin only as the control. All nanocomposites showed antifungal activity with both MIC and MFC of 250 μg/ml (Zhang et al., 2017). Following exposure to the nanocomposites, the cell surface of *C. albicans* cells were observed to have pits and perforations on the cell surface. In addition, confocal laser scanning microscopy (CLSM) showed antibiofilm activity with significantly fewer fungal cells attached to the PMMA nanocomposite surface. Cytotoxicity assays using human dental pulp cells (HDPCs) showed these nanocomposites to be nontoxic with a cell viability >75% (Zhang et al., 2017). The antifungal effect of the (AgBr/NPVP)-modified PMMA nanocomposite is dependent on the amount of AgBr/NPVP composite present and is due to the leaching of Ag^+^ which damage the fungal cell wall.

Polylactic acid (PLA) is a biocompatible polymer often used in medical implants such as mesh, screws, plates and rods as it produces lactic acid as a breakdown product. PLA electrospun nanofibrous membranes (EFMs) were developed with AgNPs (<0.1%) and cellulose nanofibrils (CNF) as antimicrobial bandages to promote ocular wound healing after trauma to the eyes (Yan et al., 2020). In-vitro cell co-culture experiments with conjunctival epithelial cells (CjECs) and circulating endothelial cells (CECs) showed excellent biocompatibility between the nanocomposite scaffold and the ocular epithelial cells. This compatibility was pronounced in the scaffolds coated with CNF where cell proliferation significantly increased (Yan et al., 2020). Importantly, these nanocomposite scaffolds also inhibited the growth of *Fusarium* spp.

### Antifungal Nanocomposites With Smooth Surfaces for Reduced Microbial Attachment

Improving the surface roughness of the material provides can also have an antimicrobial effect by reducing microbial growth sites. Fouda et al., 2019 introduced antifungal properties to PMMA for use as a dental filler by smoothening the surface of the resin with nanodiamonds (ND). This is similar to the work by De Matteis et al., 2019, where improved surface roughness leads to a reduction in *C. albicans* attachment. Fouda et al., 2019 added biocompatible NDs at various concentrations (0, 0.5, 1, 1.5% by wt) to PMMA to observe changes in *C. albicans* adhesion. The surface roughness was measured with a profilometer and the contact angle with a goniometer. The addition of NDs to PMMA smoothed the surface of PMMA and diminished the presence of peaks and valleys. This decreased the attachment of C. albicans cells on the PMMA surface due to loss of settling sites compared to the control group, with the lowest attachment observed at 1% NDs (Fouda et al., 2019).

### Antifungal Nanocomposites With Nano Encapsulated Antifungals

Another approach to develop new treatments is to improve existing antifungal drugs to overcome the associated problems of insolubility and drug penetration in human medicine. Therefore, nano encapsulated versions of these drugs are being explored. The antifungal potential of selenium and ketoconazole nanoparticles loaded into hyaluronic acid (HA) hydrogels to treat seborrheic dermatitis (SD) was evaluated by Garg et al., 2020. The antifungal mechanism of ketoconazole is well known where it inhibits the production of ergosterol, an important sterol of fungal cell membranes (Campoy and Adrio, 2017). Meanwhile the antifungal mechanisms of selenium are not as clear. However, it is thought to enhance the production of ROS, with an increase in the level of superoxide radicals resulting in fungal cell deaths (Tran et al., 2009). A hydrogel formulation loaded with ketoconazole and selenium nanoparticles by Garg et al. (2020) showed enhanced solubility, synergy and permeability of the drugs through *ex vivo* skin. Subsequently, it showed improved antifungal and anti-inflammatory activities, compared with the free drug and nanoparticle suspensions. Another advantage of NP loaded hydrogels is controlled drug release over an extended period. Thus, it allows for a maintained drug level over a specific period at the target site to enhance efficacy whilst reducing adverse side effects. HeLa cells exposed to the hydrogel formulation showed significantly higher percentage of viable cells (∼ > 90%) compared to free drugs. (Garg et al., 2020). The protective effects of the hydrogel may be attributed to HA maintenance of cell hydration as it is a component of the extracellular matrix (Yahya et al., 2014).

### Antifungal Nanocomposites Comprising Essential Oils

Essential oils (EOs) are volatile oils extracted from plants. Several EOs have been shown to have excellent antimicrobial activity and there is evidence that they can promote wound healing (Almeida et al., 2016; Saporito et al., 2017; Mahboubi et al., 2018; Labib et al., 2019). The antifungal mechanism of EOs remains unclear but is often attributed to small molecule actives within the oils. The composition of actives in these oils varies depending on the site of extraction e.g. roots or leaves. EOs studied for their antifungal activity are thyme oil (thymol and carvacrol), tea tree oil (terpenes) and peppermint or clove oil (D’agostino et al., 2019). Several studies indicate that the fungal cell wall and membrane are targets of EOs causing loss of membrane integrity and a decrease in the cell membrane ergosterol composition (de Oliveira Lima et al., 2017; D’agostino et al., 2019; Shreaz et al., 2016).

EOs show good biocompatibility when incorporated into materials such as those used in wound dressings and food packaging materials. The use of biopolymers in food packaging has increased recently due to low costs, biodegradability and edibility (Puscaselu et al., 2019). As these polymers have applications in food packaging, natural antifungal biocides such as EOs are being explored to prevent food spoilage. In some cases, the addition of these polymers to EOs also protects EOs against oxidation and evaporation (Hong and Park, 1999).

Starch edible nanocomposite films loaded with Mexican oregano (Lippia berlandieri Schauer) essential oil (MOEO) were evaluated by Aguilar-Sánchez et al. (2019) to characterise their physical and antifungal properties. These nanocomposites were formulated using MOEO (0, 1, or 2% v/v) with bentonite or halloysite (2%) and the antifungal activities against A. niger, *Fusarium* spp. and Rhizopus spp. were evaluated. A. niger showed the greatest susceptibility to the antifungal effects of the MOEO at 1% with both bentonite and halloysite nanocomposites. Whereas *Fusarium* spp. showed the most resistance with growth inhibition only observed with 2% MOEO, 2% bentonite nanocomposite (Aguilar-Sánchez et al., 2019). The nanocomposite starch films showed improved physical and antifungal properties where the addition of MOEO decreased pore density and increased pore size of the films (Aguilar-Sánchez et al., 2019).

EOs have also been observed to enhance the antimicrobial activity of antimicrobial polymers (Namivandi-Zangeneh et al., 2019). Clove oil (CO) shows antifungal activity against *Candida spp.* However, the antifungal activity of CO was greatly improved upon nano emulsification. Nanoemulsions of clove oil and pluronic F-127 with size <40 nm were produced by ultrasonic processing (Betzler de Oliveira de Siqueira et al., 2019). The MICs obtained for the nanoemulsions against *C. albicans* were 327 ± 154.15 μg/ml compared to 2,180 μg/ml for free CO (Betzler de Oliveira de Siqueira et al., 2019). The improvement in MICs may be due to improved interactions between the EO actives of the nanoemulsions and fungal cells. Eugenol, the major component of CO is thought to exert its antifungal mechanism via membrane and cell wall damage (Jafri, Ansari and Ahmad, 2019) and these effects could be facilitated by inclusion with polymers that are electrostatically attracted to fungal cells and membranes.

Coarse EO (thyme-oregano, thyme-tea tree and thyme-peppermint) emulsions were made using lecithin and tween-80 as emulsifiers. Nanoemulsions with sizes < 100 nm derived from the coarse emulsions were incorporated into chitosan films reinforced with cellulose nanocrystals (CNCs) (Hossain et al., 2019). The nanocomposite films showed significant antifungal activity against *A. nige*r, *A. flavus*, *A. parasiticus*, and *P. chrysogenum*, causing 51–77% growth reduction on inoculated rice (Hossain et al., 2019). The nanocomposite films made with the thyme-oregano EOs showed the most potent antifungal activity. The films also showed a significant increase in antifungal activity and tensile strength following exposure to 750 Gy of ionizing gamma radiation (Hossain et al., 2019).

Like other essential oil actives, thymol, a major component of thyme EO, is thought to provide antifungal effects through cytoplasmic membrane damage in addition to membrane protein interactions and various intracellular targets (D’agostino et al., 2019). It also potentially induces peroxidation of cell membrane lipids due to ROS production (Gao et al., 2016).

An advantage of nanocomposite films is its slow release of volatile components and antifungal actives over time. It has previously been observed that the incorporation of cellulose nanocrystals into the chitosan matrix increases the EO stability and also creates a diffusion matrix for slow release of EOs throughout storage (Boumail et al., 2013; Deng et al., 2017; Hossain et al., 2019). In summary, as shown in Table 4, antifungal nanocomposites show great applications in both medicine and agriculture where they can improve the structure and antifungal properties of materials.

**TABLE 4:** Summary table of antifungal nanocomposites and their reported targets.

Class	Antifungal nanocomposite	Membrane	Cell wall	Intracellular	Extracellular	Toxicity	References
Antimicrobial compounds	Nano diamonds in PMMA	—	—	—	Alters surface to inhibit fungal cell attachment	Low	Fouda et al. (2019)
	Ketoconazole selenium NPs in Hyaluronic acid gel	reduction in ergosterol production	—	ROS production and Cytochrome p450 inhibition	—	Low	Garg et al. (2020)
Metal ions	Ag^+^ based nanocomposites	Cell membrane damage	Cell wall damage	mitochondria, chromatin and ribosome	—	Low	Jia et al., 2020, Alghuthaymi et al., 2020, Zhang et al., 2017, Yan et al., 2020, De Matteis et al., 2019, Xia et al., 2016
Essential oils	Mexican oregano EO, Thyme, oregano, tea tree and peppermint EO	Decrease in ergosterol and altered membrane integrity	Cell wall damage	Endoplasmic reticulum stressor to induce the unfolded protein response, mitochondrial damage, ROS production	—	Low	Aguilar-Sánchez et al., 2019, Hossain et al., 2019
							D’agostino et al. (2019)

## Conclusion

Antimicrobial polymers show excellent antifungal activity, are cheap to synthesise, easily chemically modified and are also stable. Subsequently, they can overcome the limitations of traditional antifungal drugs as well as antimicrobial peptides. However, the exact antifungal modes of action remain unclear. Although existing antimicrobial polymers show significant antifungal properties, further research is required to improve the fungal selectivity. Polymers with innate antifungal activity are characterised by cationic regions that allow for membrane adhesion and in some cases, intracellular nucleic acid binding, hydrophobic regions that allow for membrane embedding and hydrophilic regions that control water solubility and subsequently, bioavailability. Therefore, a balance must be struck between these properties as greater regions of hydrophobicity can also lead to increased toxicity.

Due to these limitations, a supplementary role of these polymers with existing antifungal drugs might be a suitable alternative. Antifungal polymers can be combined with other antimicrobial compounds to enhance antifungal activity. Additional compounds include antifungal drugs, charcoals, lipids or metal ions which can be developed into NPs and incorporated into a range of nanocomposite materials such as creams, packaging materials or surface coatings depending on their downstream applications. This flexibility provides great promise for applications that range from postharvest food preservation to healthcare. Thus, the potential of antifungal polymers to replace antifungal drugs remains.
